# Direct Growth of MoS_2_ Nanowalls on Carbon Nanofibers for Use in Supercapacitor

**DOI:** 10.1038/s41598-017-05805-z

**Published:** 2017-07-20

**Authors:** Fitri Nur Indah Sari, Jyh-Ming Ting

**Affiliations:** Department of Materials Science and Engineering, National Cheng Kung University, Tainan, Taiwan

## Abstract

Direct growth of MoS_2_ nanowalls on vapor grown carbon nanofibers (VGCNFs) has been achieved using a microwave-assisted hydrothermal (MAH) method under an acidic condition. The acidic condition was obtained through the addition of an HCl aqueous solution. We demonstrate that the HCl not only modifies the pH value for limiting the growth rate but also leads to the formation of NaCl, which is the key for the direct and unique growth of MoS_2_ on the VGCNF surface. A growth mechanism is therefore proposed. The growth of MoS_2_ onto the high electrically conducting VGCNF creates a unique structure that not only reduces the aggregation of MoS_2_ but also improves the electrical conductivity of the resulting composite electrode. The MoS_2_ nanowall/VGCNF composite shows Csp as high as 248 F g^−1^ at 5 mV s^−1^ and excellent electrochemical stability with a retention of 96% after 1,000 cycles at a high charge rate of 200 mV s^−1^. The ease of composite fabrication and electrochemical stability suggest that the MoS_2_ nanowall/VGCNF composite is a promising candidate electrode material for supercapacitor.

## Introduction

Recently, two-dimensional (2D) materials, such as graphene, MXenes (a group of early transition metal carbides or carbonitrides), and transition metal dichalcogenide (TMD) are attracting a lot of attentions due to their unique structures that are desirable for many applications, such as energy storage devices. Graphene has been investigated by many researchers for supercapacitor application^1–3^. MXenes, due to the difficulties in the fabrication of the precursor materials, i.e., MAX phase (M is an early transition metal, A is a group 13 or 14 element, and X is C and/or N), and the subsequent needed etching process, have found themselves limited development. It therefore appears that TMD materials, such as MoS_2_, WS_2_, and SnS_2_, are becoming a major part of the main stream. These metal sulphide materials have a structure analogous to that of graphene where S–M–S (M = Mo, W, Sn, etc) covalent bonds exist within the basal planes and van der Waals forces hold the planes^4^. Among these TMD materials, earth abundant MoS_2_, exhibiting high hydrogen evolution reaction (HER) activity, large surface area, and 2D structure favouring ion adsorption and transport^5^, has found itself various applications including solar cell, Li-ion battery, Na-ion battery, and electro catalysis for HER^5–9^. Recently, MoS_2_ has also evolved as an attractive supercapacitor electrode material^10, 11^. Its graphene-like morphology and large surface area are desirable for such an application. The charge storage mechanisms of MoS_2_ include (i) inter-sheet electrical double layer capacitance (EDLC), (ii) intra-sheet EDLC between individual atoms of MoS_2_, and (iii) faradaic charge transfer on the Mo centers due to the various oxidation states (+2 to +6) of Mo^12^. However, the faradaic charge transfer is not always observed during the process. It depends on the cation size of the electrolyte and mostly occurs at a very low scan rate ~1 mV s^−1^ ^12^. For example, among the alkali cations of Li^+^, Na^+^, and K^+^ having solvated ion size of 6, 4, and 3 Å, respectively, only the smallest K^+^ ions give distorted but not rectangular CV curve due to their intercalation into the MoS_2_ layers^13^. Also, obvious redox peaks commonly appear due to the easiness of H^+^ intercalation into MoS_2_ layers^13, 14^.

Despite of these prominent features, the practical application of MoS_2_ as an electrode of supercapacitor still faces challenges due to its low intrinsic conductivity that leads to low energy density^7, 10, 12^. Hence, several means have been explored to overcome these drawbacks, such as by adding a carbon material and/or conducting polymer. It has been reported that carbon can improve the electron transport in nanostructured MoS_2_ electrode and thereby produce enhanced capacitance^15, 16^. Up to now, the carbon materials that have been used as a conductive additive in MoS_2_ are graphene and it derivatives. In a 3D sphere-like MoS_2_/reduce graphene oxide (RGO) composite, the RGO serves as a conductive network that facilitates the electron transport. The 3D structure provides pores that serve as ion reservoirs for ions intercalation within the electrode material^16^. RGO has also been used as a template to grow MoS_2_^15^. It was found that more RGO than MoS_2_ is needed in order to form few-layered but not multiple stacked-layered MoS_2_. This allows the composite to give enhanced inter-and intra-sheet charge storages and better retention (92% after 1,000 cycles). MoS_2_/N-doped graphene (NDG) composite has been synthesized using a hydrothermal process in which the growth of MoS_2_ nanosheets and the formation of NDG occur in the same pot^11^. The NDG contains N- and O-functional groups which improve the wettability of the electrode surface and allow additional faradaic reactions, hence leading to enhanced pseudocapacitance. However, graphene or its derivatives are easily to stack or re-stack and aggregate. Therefore, there is a need of surface modification to prevent these phenomena. On the other hand, the addition of a conducting polymer, especially polypyrrole (PPy) and polyaniline (PANI), into MoS_2_ for enhanced electrical conductivity is also reported^10, 17^. However, the major drawbacks of the resulting composites include poor cyclic life due to the irreversible redox reactions and highly undesirable degradation of the composite during the charge-discharging process.

As mentioned above, MoS_2_ has been mixed with graphene or its derivatives, however, the growth of MoS_2_ on a carbon material is not direct. The contact between a carbon material and MoS_2_ is limited, leading to large interface resistance. Carbon is normally negatively charged^18^ and so is MoS_2_ in acid condition^19^. Under such as circumstance, the use of surfactant or the existence of some functional groups on the carbon surface is required to improve the interfacial contact between MoS_2_ precursor and carbon. Up to now, several approaches have been taken. Glucose was used to act as a binder to help the interaction between MoS_2_ precursor and carbon^20–22^. Cationic surfactant (cetyltrimethylammonium bromide) modifies graphene surface through electrostatic interaction, thus reducing the charge incompatibility between the graphene and the MoS_2_ precursor^23^. Also, gemini surfactant homogeneously functionalizes the surface of GO so that the wettability of GO is improved^24^.

In this work, we demonstrate the synthesis of MoS_2_ nanowalls on a carbon material, namely, vapour grown carbon nanofiber (VGCNF), without the use of any binder, surfactant, or surface modification. MoS_2_ nanostructures especially nanoflower-like MoS_2_ have been synthesized using the conventional hydrothermal technique^5, 25–28^. However, this approach requires a long reaction time ranging from 15 to 48 hours. Here we have used a facile microwave-assisted hydrothermal (MAH) method for the synthesis of MoS_2_ nanowalls in a just few minutes under an acidic condition. The pH value plays an important role in controlling the growth of MoS_2_, for example, few-layered MoS_2_ exposing plenty active sites was obtained under an acid condition that reduced the MoS_2_ growth rate^29^. The acidic condition was achieved through the addition of an HCl solution. However, we show that the HCl in fact not only modifies the pH value but also leads to the formation of NaCl in the solution. The formation of NaCl is desirable since it improves the hydrophilicity of the carbon nanofiber by providing additional O-functional groups and hence makes the surface charge to become more positive^30, 31^. Therefore, we demonstrate that the presence NaCl on the surface of the VGCNF allows the growth of MoS_2_ on the carbon nanofiber. Furthermore, to our knowledge, MoS_2_/VGCNF composite for use as a supercapacitor electrode has not been reported. VGCNF is a highly graphitic carbon, exhibiting excellent thermal and electrical conductivity^32–35^. As a result, the growth of MoS_2_ on VGCNF creates a unique structure that not only reduces the aggregation of MoS_2_ but also improves the electrical conductivity of the resulting electrode.

## Results and Discussion

### Structure and morphology of MoS_2_ nanowall/VGCNF composites

**Figure 1:**
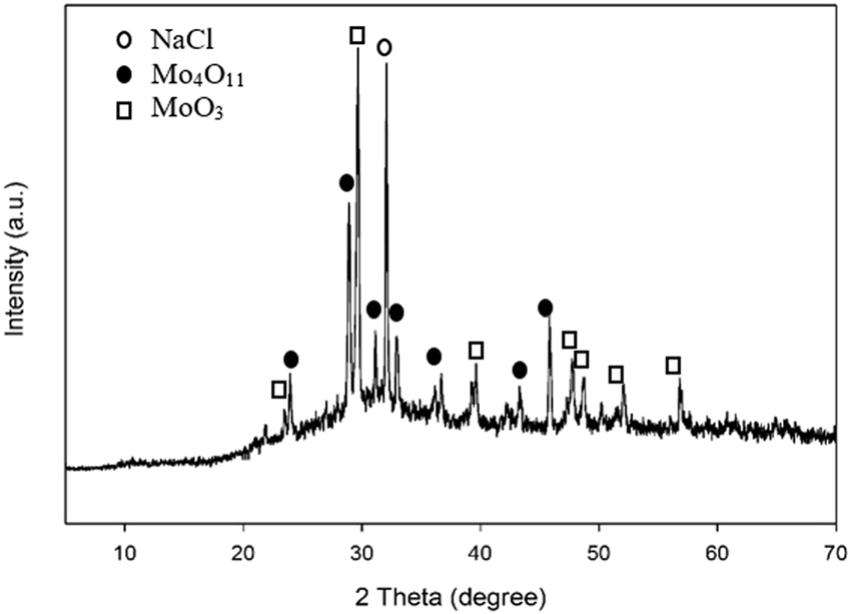
XRD pattern of solid precursor before the MAH treatment.

The synthesis of MoS_2_/VGCNF composites was conducted through a simple MAH method. Prior to the MAH treatment, the solution was found to be milky. After the solution was dried, the obtained solid precursor sample was subjected to XRD analysis. The resulting XRD diffraction pattern shows that the solid precursor consists of NaCl, Mo_4_O_11_, and MoO_3_ (Fig. 1). The milky appearance is therefore due to the formation of the Mo oxide particles as shown in Figure S1 in the Supporting Information. Before the MAH treatment, the HCl in the precursor solution triggers the hydrolysis of Na_2_MoO_4_^.^2H_2_O and leads to the formation of NaCl and MoO_3_ as expected. However, it is believed that during the formation of the stable MoO_3_, sub-oxide Mo_4_O_11_ appears as an intermediate, minor phase due to the partial reduction of the Mo^6+^ in Na_2_MoO_4_. During the MAH synthesis, interaction between the H^+^ ions and the MoO_3_ causes such reduction, hence forming Mo_4_O_11_. The occurrence of Mo_4_O_11_ during the reduction of MoO_3_ has been observed elsewhere^36, 37^. It is noted that, based on the XRD peak areas, the amount of the Mo_4_O_11_ was found to be about 40% that of the MoO_3_. Also, the CH_4_N_2_S reacts with H_2_O and form H_2_S gas^10^. H_2_S gas is known to react with MoO_3_ to form MoS_2_ according to Eq. 1A ^7^. In the meantime, we believe that the Mo_4_O_11_ also reacts with H_2_S in a similar fashion as shown in Eq. 1B.

1A $$4{{\rm{MoO}}}_{3}+9{{\rm{H}}}_{2}{\rm{S}}=4{{\rm{MoS}}}_{2}+{{\rm{SO}}}_{3}+9{{\rm{H}}}_{2}{\rm{O}}$$

1B $$4{{\rm{Mo}}}_{4}{{\rm{O}}}_{11}+35{{\rm{H}}}_{2}{\rm{S}}=16{{\rm{MoS}}}_{2}+3{{\rm{SO}}}_{3}+35{{\rm{H}}}_{2}{\rm{O}}$$

Although the above reactions explain the formation of MoS_2_ itself, as mentioned above, the growth of MoS_2_ on a carbon material is not straight forward. The formed NaCl prior to the MAH process plays a crucial role that allows the direct growth of MoS_2_ on VGCNFs. It was found that the zeta potentials of individual VGCNFs and MoS_2_ solid precursors are −56.6 and −15.7 mV, respectively. The negatively charged species hence repel each other. However, during the MAH synthesis, the presence of NaCl in the solution modifies the surface of VGCNF by giving various O-functional groups^30^. As to be shown later, O-functional groups such as -OH and -COOH pair with the carbon lattice on the VGCNFs surface such that the wettability the Mo radicals on the carbon nanofibers is increased, hence allowing the growth of MoS_2_ on the VGCNFs. Furthermore, a recent report shows that NaCl changes the surface charge of graphene oxide from negative to positive^31^. In this study, the critical role of NaCl will be discussed later. Also, with a higher molar ratio of Mo precursor, more MoS_2_ was formed not only heterogeneously on the VGCNFs but also homogeneously by the VGCNFs. The detailed morphology will be explained with the SEM analysis given below.

**Figure 2:**
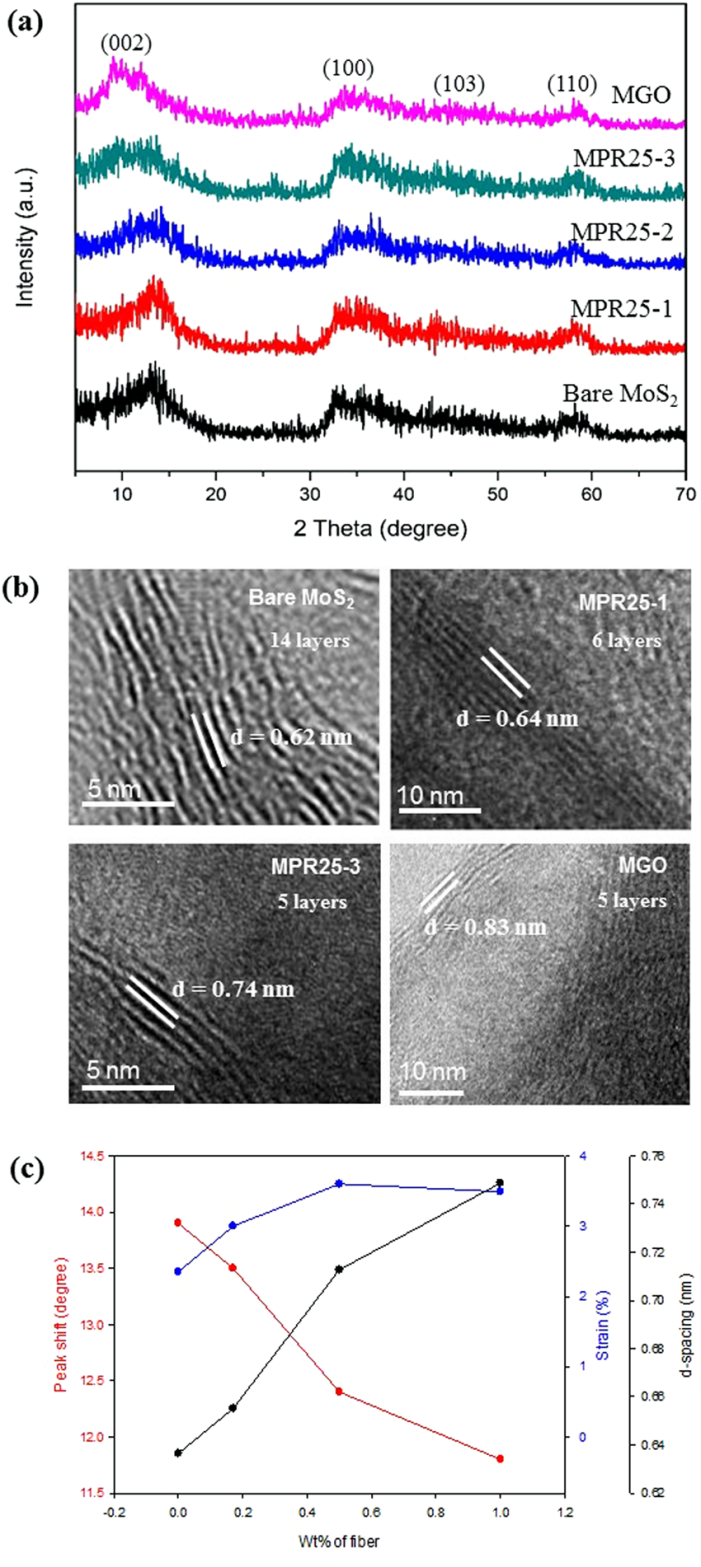
(**a**) XRD diffraction patterns, (**b**) HR-TEM images of bare MoS_2_ and its composites and their d-spacing and no. of layer, and (**c**) effect of the wt% of fiber to the peak shift, strain, and d-spacing.

The crystalline phase was determined using XRD. All the composites show the hexagonal phase of MoS_2_ (JCPDS 37-1492) with several major diffraction peaks at 12.9°, 33.3°, 37.6°, and 58.2°, corresponding to the (002), (100), (103), and (110) planes of MoS_2_, respectively (Fig. 2a). There is no other peak observed in the XRD patterns, indicating the phase purity of the obtained MoS_2_. No carbon peak was detected due to the small amounts of VGCNFs, except that a carbon peak at 26.4° was detected for MPR25-3 which has the highest amount of VGCNFs. It is noted that there is no peak at 14.4° indicating the formation of partially exfoliated MoS_2_^38^. We can infer that the MoS_2_ in the composites consists of a few layers which can be observed obviously using TEM analysis to be shown later. However, it is seen that the intensities of peaks are low and the peaks are broadened, indicating poor crystallinity of the MoS_2_. With increasing VGCNFs, the peak at 12.9°, corresponding to the MoS_2_ (002) plane diffraction, becomes more broadened. The full width half maximum (FWHM) are 3.7, 3.2, 5.2, and 7.3 for MoS_2_, MPR25-1, -2, and -3, respectively. It is believed that the presence of VGCNFs restrains the growth of layered MoS_2_ crystal during MAH synthesis especially the (002) plane^11, 16^, hence resulting in reduced crystal size of MoS_2_. Furthermore, the (002) peak shifts to the smaller angle side after the addition of VGCNFs. Such a peak shift is more pronounced for Sample MGO, whose (002) peak shifts to an even lower angle of 10.4° (Fig. 2a). The peak shift indicates both the expansion of the d-spacing and the existence of strains^25^. The expansion is obviously as shown in the HR-TEM images given in Fig. 2b. Accordingly, the interlayer distances of bare MoS_2_, MPR25-1, and MPR25-3 are 0.62, 0.64, and 0.74 nm, respectively. We also believe that strains exist in the obtained MoS_2_ on the VGCNFs. This accounts for the different in the d-spacing determined from the XRD and TEM (Table S1). The d-spacing determined from the XRD are larger than that determined from the HRTEM images. This is attributed to the existence of strains. Such strains can be estimated using the Wagner-Agua method^39^. The value of the strain increases with the amount of fibers (Fig. 2c). Furthermore, the addition of VGCNFs results in significant decrease of the number of MoS_2_ layers as shown in the HR-TEM images given in Fig. 2b. For example, the number of layers significantly decreases from ~14 to ~6 layers for MoS_2_ and MPR25-1, respectively.

**Figure 3:**
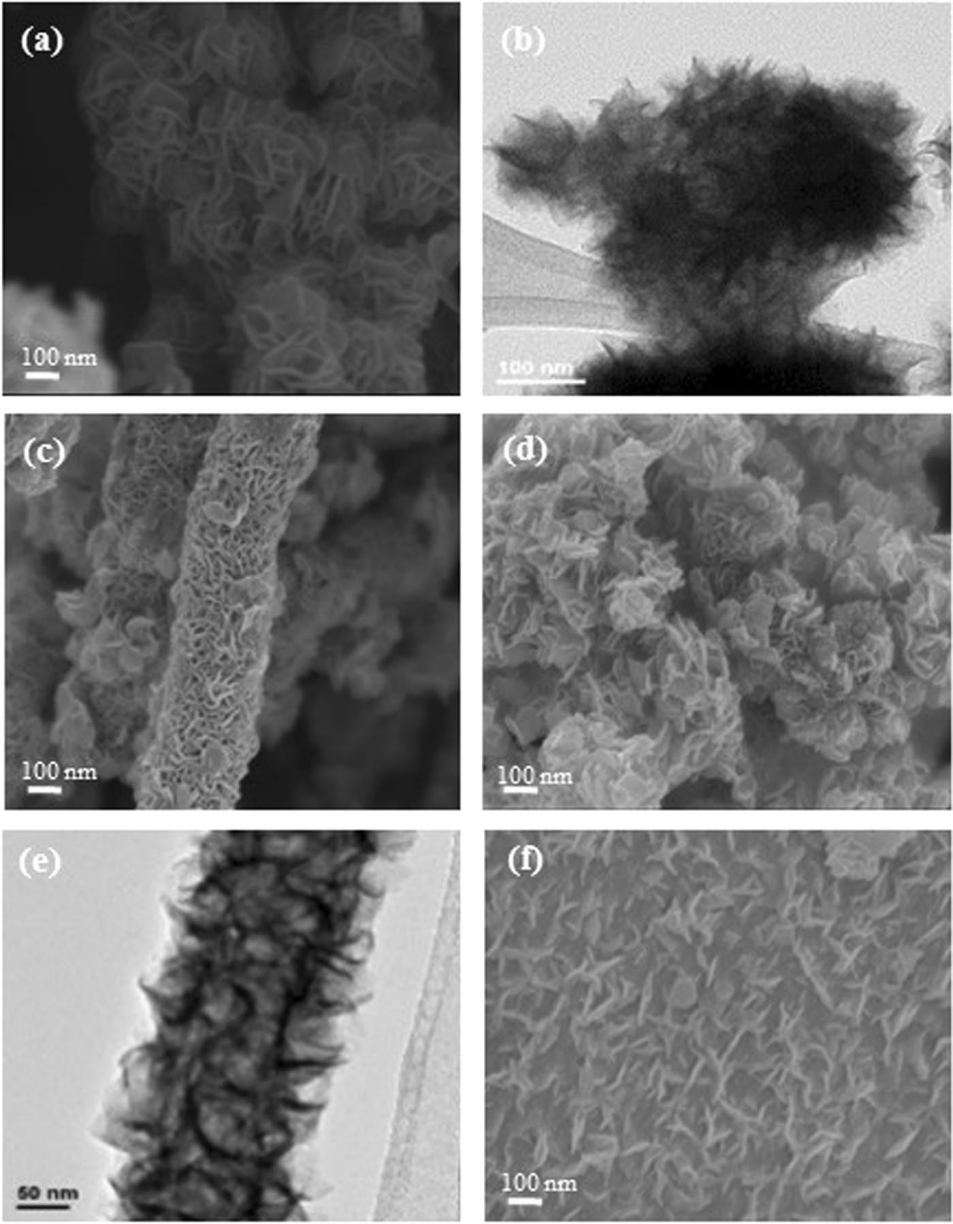
(**a**,**b**) SEM image and HR-TEM image of bare MoS_2_, (**c**,**d**) SEM images of MoS_2_ on the fiber surface and by the fiber for MPR25-2, respectively, (**e**) TEM image of MoS_2_ on the fiber surface for MPR25-3, and (**f**) SEM image of MoS_2_ on the RGO surface.

Figure 3 shows the morphologies of the bare MoS_2_ and its composites. The MoS_2_ exhibits a flower-like morphology having MoS_2_ nanowall petals (Fig. 3a,b). It is known that the formation of MoS_2_ nanoflowers begins with the occurrence of MoS_2_ nanowalls which then aggregate, due to the van der Waals interaction, and then form a flower-like morphology^40^. However, the growth of MoS_2_ on a VGCNFs surface involves more effort as mentioned above. In the composite samples, MoS_2_ nanowalls and nanoflowers were found on the fibers surfaces (Fig. 3c) and by the fibers (Fig. 3d), respectively. The amount of MoS_2_ nanoflowers by the VGCNFs in general increases with the Mo precursor. The growth of MoS_2_ nanoflowers by the fibers follows the mechanism describe above. On the other hand, the growth of MoS_2_ nanowalls on the fibers surface in this study is not straightforward but due to the addition of NaCl which changes the polarity of VGCNFs surface and gives O-functional groups. Hence, under such a circumstance, heterogeneous growth of MoS_2_ nanowalls on the fibers surface occurs. When the amount of Mo precursor exceeds a certain amount, the homogeneous growth of MoS_2_ nanoflowers by the fibers also occurs. It appears that the nanowalls are vertical to the fiber surface, as shown in Fig. 3e. Figure 3e also shows that each nanowall consists of a few stacked layers of MoS_2_. The contact between the MoS_2_ nanowall and fiber surface is intimate. Furthermore, with increasing fiber, the nanowalls become thicker and their packing becomes denser, as shown in Figure S2. It shows that compared to MPR-3, MPR25-1 nanowalls are thinner, approximately 22 nm, and loosely packed. MPR25-2 (Fig. 3c) and -3 (Figure S2) show denser packing of the nanowalls whose thicknesses are thicker too, approximately 25 nm and 30 nm, respectively. Thinner nanowalls and less packing density give more empty spaces in between the nanowalls which is beneficial for ion intercalation. For comparison, MoS_2_ was also grown on GO, which was reduced to RGO during synthesis. Layers of MoS_2_ were found to grow on the RGO surface, laying parallel to the RGO surface (Fig. 3f), while MoS_2_ nanoflowers are seen beside the RGO (Figure S3). The fact that MoS_2_ grows on either the fiber or RGO surface demonstrate the critical role of NaCl, which facilitates the nucleation and growth of MoS_2_, as to be discussed more later. To further investigate the growth mechanism, a control experiment has been done following the condition for MPR25-1, except without the sonication. The sonication helps to disperse the precursors such that the nucleation is more homogeneous and therefore the MoS_2_ nanowalls also distribute more homogeneously, as shown in Figure S4.

**Figure 4:**
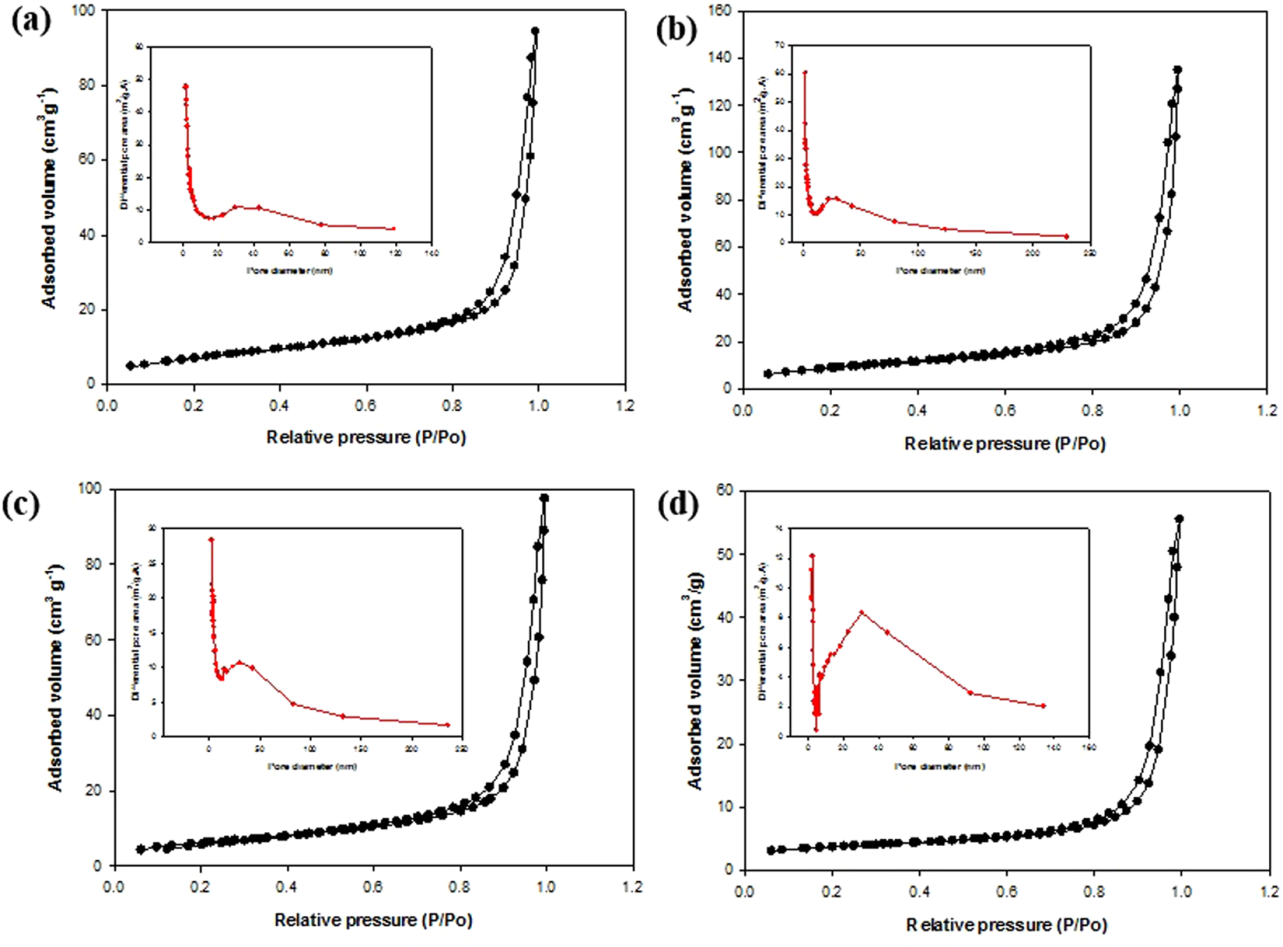
N_2_ adsorption-desorption of (**a**) bare MoS_2_, (**b**) MPR25-1, (**c**) MPR25-2, and (**d**) MPR25-3. The insets show pore size distributions obtained using the BJH method.

The difference in the morphology results in different SSA, as shown in Table 1. It shows that the BET SSA of MPR25-1 > bare MoS_2_ > MPR25-2 > MPR25-3 > MPR25-1-2. Initial addition of fiber to the MoS_2_ increases the SSA of the resulting MPR25-1 composite from 26.98 to 32.26 m^2^ g^−1^. However, further increasing the fiber amount (MPR25-2 and -3) leads to decreased SSA due to the thicker and more densely packed nanowalls, as mentioned above. This also indicates that the MoS_2_ has a higher SSA than the VGCNF. Likewise, MPR25-1-2 has smallest SSA among the composites due to the vigorous aggregation of the nanowalls. The result strongly suggests that the introduction of an appropriate amount of VGCNFs can inhibit the aggregation of MoS_2_ and will be further explained later in the mechanism part. Compared to MPR25-1, MGO has a much smaller SSA because the RGO layers are easy to stack or restack. The nitrogen adsorption-desorption isotherms of bare MoS_2_ and its composites exhibit type IV characteristics which indicates the existence of large pores or mesopores (Fig. 4). Using the BJH method the pore size distributions were characterized, as shown in insets of Fig. 4. It can be clearly seen that MPR25-1 has the largest pore volume (Table 1). It is known that the flower-like morphology has larger pore size than the wrinkle layered morphology. Therefore, as shown in Table 1, bare MoS_2_ has the highest pore size and the composites have similar pore sizes too. However, among all of the samples, MGO has the highest pore size. This is attributed to the existence of MoS_2_ by the RGO that exhibits a fully 3D flower-like structure, as shown in Figure S3.

**Table 1:** BET and BJH analysis of bare MoS_2_ and composites.

ID Sample	SSA (m^2^ g^−1^)	Pore Volume (cm^2^ g^−1^)	Pore Size (nm)
Bare MoS_2_	26.98	0.049	36.23
MPR25-1	32.26	0.165	29.45
MPR25-2	22.48	0.117	29.99
MPR25-3	12.55	0.029	30.39
MPR25-1-2	6.26	0.01	31.78
MGO	3.2	0.032	40.57

**Figure 5:**
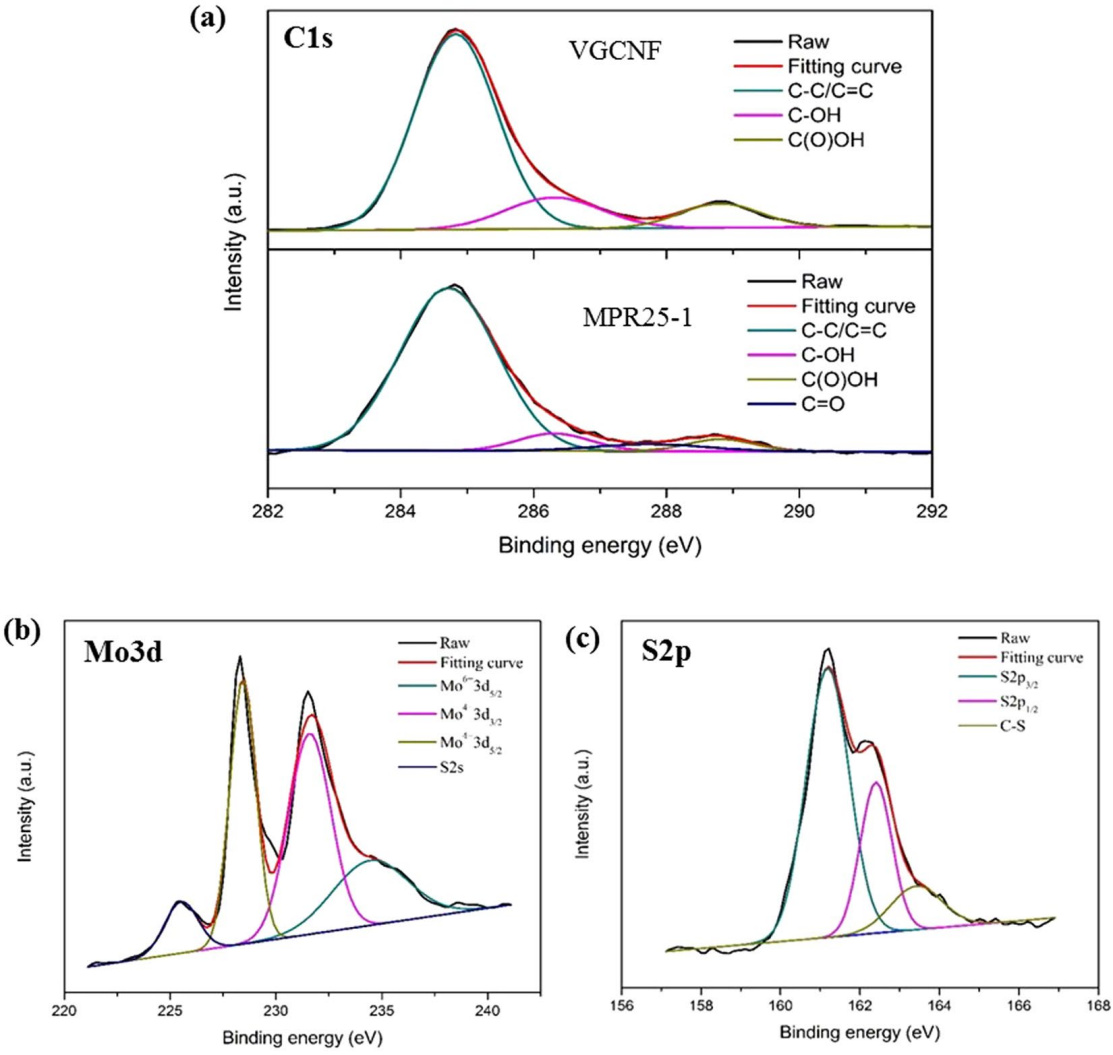
XPS spectra of (**a**) C1s for VGCNF and MPR25-1, (**b**) Mo3d for MPR25-1, and (**c**) S2p for MPR25-1.

As mention above, the presence of NaCl on the VGCNF surface is the key for the growth of MoS_2_ on the fiber. It is known that in H_2_O, NaCl is completely dissolved due to the polar nature of H_2_O. As a result, the electrostatic force between Na^+^ and Cl^−^ breaks, leading to the formation of their solvated ions. Furthermore, it is known that the interaction between Na^+^ and π-electrons is stronger than that between Cl^−^ and π-electrons, therefore the solvated Na^+^ is attracted to the fiber surface by the π-electrons on the carbon fiber^41^. Under such a circumstance, the fiber surface becomes positively charged due to the existence of the Na^+^ and the solution becomes negatively charged due to the presence of Cl^−^. In the meantime, during the hydrothermal process, the solvated Na^+^ that are attracted to the fiber surface react with the C-C/C=C and O-functional groups on the fiber surface to form C=O bonding. The existence of C=O bonding is supported by the high resolution XPS C1sp spectra of VGCNF and MPR25-1, as shown in Fig. 5a. XPS C1s spectra of the rest of the composites are shown in Figure S5. Three common peaks are seen in both spectra. The dominant peak at 284.7 eV is attributed to graphitic carbon C-C/C=C, while the other two peaks at 286.3 and 288.8 eV are related C-OH and C(O)OH respectively^26, 42^. However, there is an additional peak of C=O at 287.7 eV in the C1sp spectrum of MPR25-1 and the rest of composites as shown in Figure S5. This indicates the aforementioned Na^+^ adsorption to and reaction on the fiber surfaced have indeed occurred. The Mo3d and S2p high resolution spectra for Sample MPR25-1 were also obtained, as shown in Fig. 5b,c, respectively. There are three Mo orbital peaks at 229.5, 232.6, and 235.9 eV, representing Mo^4+^3d_5/2_, Mo^4+^3d_3/2_, and Mo^6+^3d_5/2_, respectively, and a S2s peak at 225.6 eV in the high resolution Mo3d spectrum. The dominant peak of Mo^4+^ is from the MoS_2_ bonding and the weak peak of Mo^6+^ is likely due to the presence of small amount of Mo-O bonding which commonly occurs due to either the bonding between Mo atom and O atom from the functional groups on the fiber or partially oxidation^11^. The S2s peak represent the Mo-S bonding. On the other hand, there are various bonding at 161.4, 162.6, and 163.7 eV, representing S2p_3/2_ and S2p_1/2_ for Mo-S and C-S bonding, respectively, in the high resolution S2p spectrum^11, 43^.

### Direct growth mechanism of MoS_2_ nanowalls on VGCNFs

**Figure 6:**
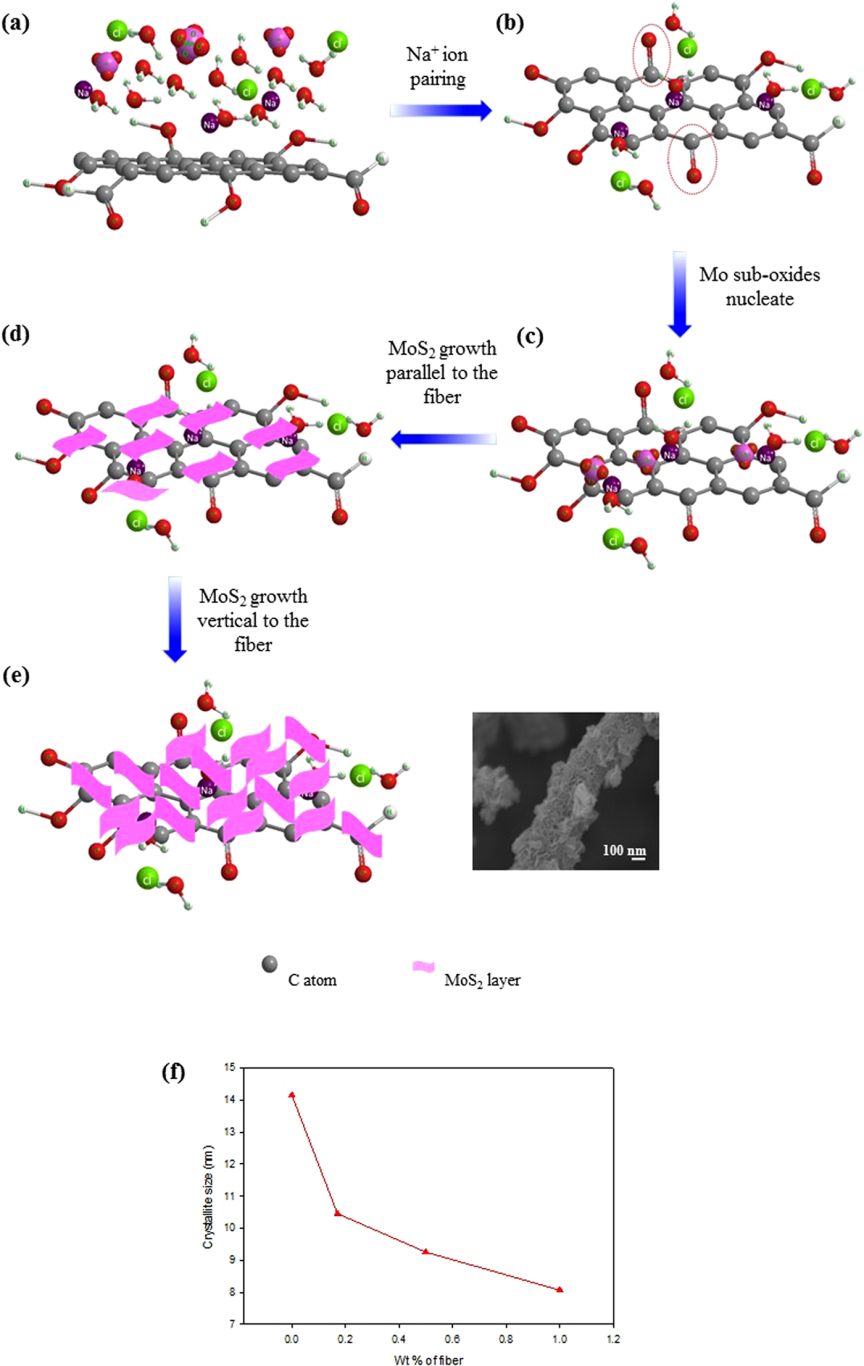
Schematic illustration growth mechanism of MoS_2_ nanowall on VGCNF assisted by NaCl. (**a**) Fiber in the solution containing solvated Na^+^ and Cl^−^, MoO_3_, and Mo_4_O_11_, (**b**) Na^+^ ion pairing to the fiber and additional carbonyl group (C=O) indicated by red circle dash line, (**c**) MoO_3_ and Mo_4_O_11_ start to attract and nucleate on the fiber surface, (**d**) MoS_2_ layer growth parallel to fiber surface, (**e**) MoS_2_ layer tend to curveting and growth vertical to the fiber and its SEM image, and (**f**) crystal size decreasing with increasing of fibers amount.

Based on the XRD, SEM, TEM, and XPS analysis, the growth mechanism of MoS_2_ on the VGCNF is proposed as shown schematically in Fig. 6. During the synthesis, there are both heterogeneous growth of MoS_2_ on the fiber surface and homogeneous growth of MoS_2_ by the fiber. As mentioned previously that the growth of MoS_2_ on the fiber surface is due to the use of NaCl. As shown in Fig. 6a, fibers in the solution are surrounded by the solvated Na^+^ and Cl^−^, MoO_3_, and Mo_4_O_11_. Due to the interaction with the π-electron from the fibers, the solvated Na^+^ are attracted to the fiber surfaces (Fig. 6b), hence making the fiber surface positive as supported by the Zeta potential measurements (Figure S8 in the Supplementary information). As a result, due to the existence of the solvated the Na^+^ on the fiber surface, C=O bonding forms during the MAH according to Eq. 2.

2 $${\rm{R}}-{\rm{CO}}({\rm{OH}})+{{\rm{Na}}}^{+}\to {\rm{R}}-{\rm{C}}={\rm{O}}+{\rm{NaOH}}$$

where R is aromatic of carbon.

Therefore, the wettability of the fibers is improved as shown in Fig. 6b. Under such a circumstance, both the negatively charged MoO_3_ and Mo_4_O_11_ are attracted to the positively charged fiber surface, as shown in Fig. 6c. During the hydrothermal process, these sub-oxides are reduced to MoS_2_ by H_2_S released from thiourea as shown in Eq. 1A and B. The single- and then few-layered MoS_2_ grow on the fiber surface due to the lowest surface energy of (002) planes of MoS_2_ as shown in Fig. 6d. The layered growth phenomena at the initial stage can be clearly seen in MGO sample as shown in Fig. 3f. As the MoS_2_ grows layer by layer to the [001] direction, more edges are exposed in the few-layered structure. The edges have different local stoichiometric as compared to the basal plane, i.e., lower coordination numbers. The unsaturated S atoms on the edge sites from either their atomic structure of H phase or structural defect represent the microstructure having low coordination numbers, hence making the edges more reactive than basal plane^44, 45^. Furthermore, the edge sites also show metallic phase and therefore accommodate ions, making them more reactive too^44^. Consequently, thermodynamic driving force makes the MoS_2_ to grow vertically to the fiber or RGO, as shown in Fig. 6e, to minimize the exposures of the highly energetic and atomically undercoordinated edges. On the other hand, a larger amount of fibers gives a lower pH, meaning more positively charged surfaces. The fibers then attract more MoO_3_ and Mo_4_O_11_, i.e., forming more nucleation sites for the growth of MoS_2_. As a result, due to the increased nucleation sites, the MoS_2_ grows to smaller sizes in the basal planes on the fiber surfaces, as shown in Fig. 6f. Also, as discussed above, strains were found in the composite samples and the strain increases with the fibers (Fig. 2c). In the meantime, a larger amount of fibers, giving a larger strain, leads to a larger d-spacing (Fig. 2b,c). The strain is interfacial strain that occurs due to the lattice mismatch between the fiber and MoS_2_. Such interfacial strain leads to uniaxial tensile strain along [001] direction that causes the expansion of the lattice along the [001] direction^46^. As a result, a larger amount of fibers, meaning a higher strain, leads to a larger d-spacing.

### Electrochemical performance of MoS_2_ nanowall/VGCNFs composites

**Figure 7:**
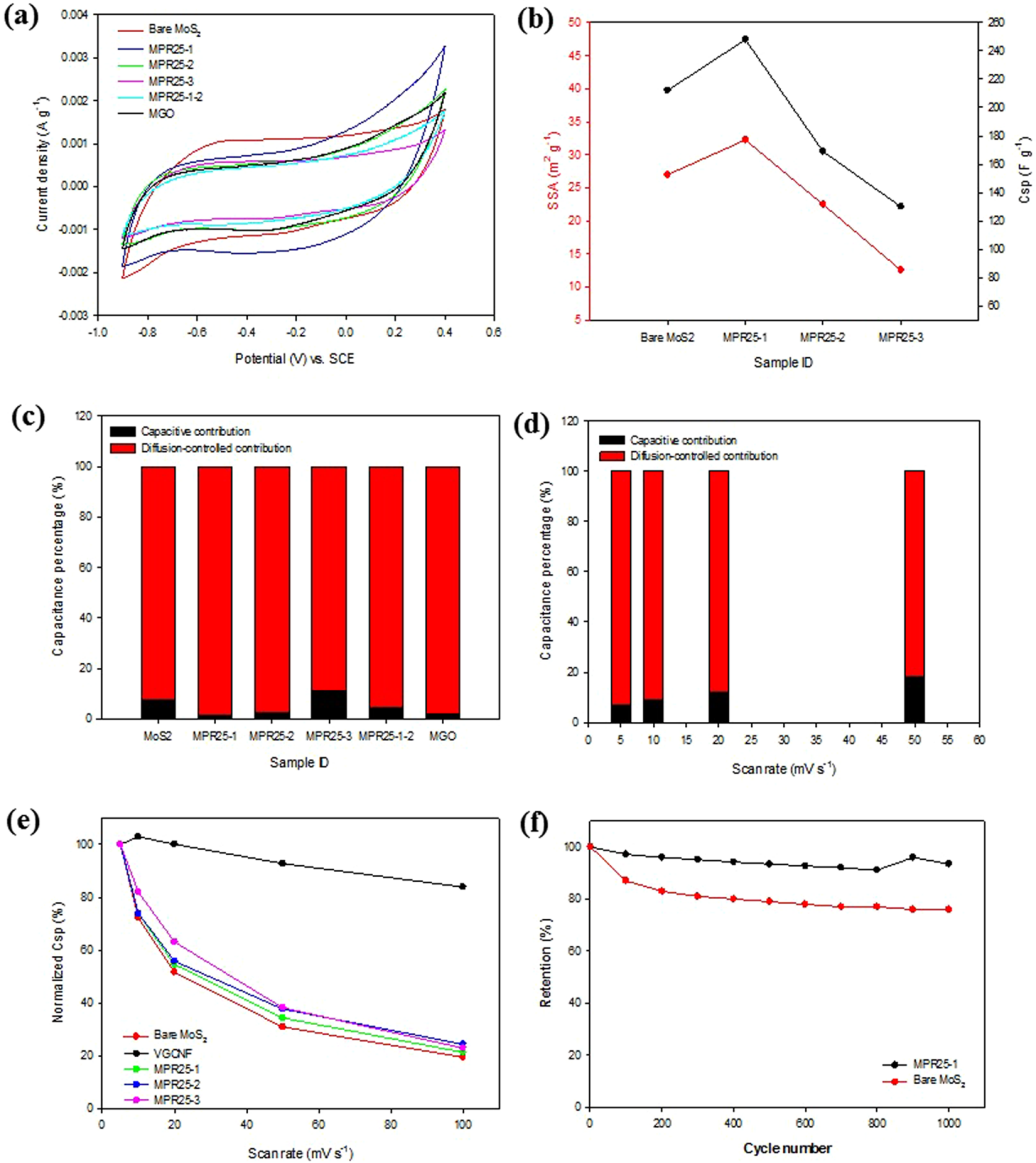
(**a**) CV curves of bare MoS_2_ and composites at 5 mV s^−1^ and (**b**) the relationship between SSA and Csp of bare MoS_2_ and composites. Charge storage calculations of (**c**) bare MoS_2_ and composites based on Trasatti’s and (**d**) MPR25-1 based on Dunn’s methods. (**e**) Normalized Csp as a function of scan rate. (**f**) Cyclic stability test of bare MoS_2_ and MPR25-1 at 200 mV s^−1^.

The electrochemical performance of bare MoS_2_ and its composites were evaluated using CV and EIS tests. Figure 7a shows the CV curves of bare MoS_2_ and its composites. The curves show quasi-rectangular shapes without any obvious redox peak, seemly suggesting the charge storage of all the samples are likely from EDLC. However, the specific capacitance determined from these CV curves is considered to be more than that can be obtained based on the consideration of only the moderate specific surface areas of the electrode materials (Table 1). Therefore, it is believed that pseudocapacitance also exists. In other words, the charge storage of MoS_2_ in this study comes from both EDLC and pseudocapacitance, which is contributed by diffusive-intercalation and surface redox reactions. Comparing all of the CV curves, MPR25-1 shows more distortion at the positive potential side. This occurs due to its higher pseudocapacitance as explained later. The Csp of all the samples were calculated by integrating the area under the CV curve. As shown in Fig. 7a, the MPR25-1 has a wider area as compared with other samples followed by bare MoS_2_ and MPR25-2 then MPR25-3. Therefore, the Csp (at 5 m V s^−1^) are in the same order: MPR25-1 (248 F g^−1^) > bare MoS_2_ (212 F g^−1^) > MPR25-2 (169 F g^−1^) > MPR25-3 (130 F g^−1^) > MPR25-1-2 (138 F g^−1^) > VGCNF (17 F g^−1^) (The CV curve of VGCNF is given in Figure S6). MPR25-1 has the highest Csp due to the highest SSA and thinnest nanowalls. Noteworthy, the distance between the layers of MoS_2_ is very important in charge transport. A sufficient distance between adjacent nanowalls prevents the electrostatic repulsion between adsorbed ions^12^. It also shows in Table 1 that MPR25-1 exhibits large pore volumes which favour ion diffusion. It indicates the presence of fiber would open the pores in the MoS_2_ by inhibiting the aggregation of the MoS_2_ layers. Comparing the Csp of MPR25-1 (248 F g^−1^) and MGO (165 F g^−1^), it is seen that the VGCNF is more favourable for the electron transport than GO. The reason is that the GO exhibit a lower electrical conductivity. However, there is limit for the addition of fiber, as shown in Table 1. The SSA and pore volume decrease with the amount of the VGCNF. Therefore, the Csp of MPR25-2 and MPR25-3 are lower than MPR25-1. Thicker and aggregated MoS_2_ nanowalls were observed for MPR25-2. Both observations are linked to less favourable electron transport. For the bare MoS_2_, it has the second highest Csp due to the high SSA and appropriate pore sizes for ion diffusion. Therefore, from the above observation, a clear relationship between SSA and Csp can be obtained, as shown in Fig. 7b for bare MoS_2_ and MoS_2_/VGCNF composites.

Here in, the charge storage mechanism is further discussed. A quantitative analysis has been done to determine the percentage of diffusion-controlled and capacitive capacitance following the Trassatti method^47^. The result shows that diffusion-controlled capacitance is the major storage mechanism for all the samples, as shown in Fig. 7c. The diffusion-controlled capacitance is believed to come from the intercalation of K^+^ into the MoS_2_ interlayers, as shown in Eq. 3.

3 $${{\rm{MoS}}}_{2}+{{\rm{K}}}^{+}+{{\rm{e}}}^{-}\leftrightarrow {\rm{MoS}}-{\rm{SK}}$$

Meanwhile, the capacitive capacitance is from double layer charges and the surface redox reaction as shown in Eq. 4.

4 $${{\rm{MoS}}}_{2}\,{\rm{surface}}+{{\rm{K}}}^{+}+{{\rm{e}}}^{-}\leftrightarrow ({{\rm{MoS}}}_{2}-{{\rm{K}}}^{+})\,{\rm{surface}}$$

Furthermore, the kinetic charge storages at different scan rates determined by the Dunn’s method^48^ also show that the diffusion-controlled is dominant, as shown in Fig. 7d for MPR25-1. The capacitive contribution increases with the scan rate. Likewise, the other samples exhibit the same behavior as shown in Figure S7. On the other hand, the addition of fibers to the MoS_2_ improves the Csp retention that favours the high power density as shown in Fig. 7e. It shows that a composite with a higher amount of VGCNF has better retention than the bare MoS_2_. Especially, MPR25-2 shows an enhancement of 25% over the bare MoS_2_ at 100 mV s^−1^, indicating the fibers help the electron transport during the charge-discharge process. These observations indicate that, for both the electrochemical characteristic of bare MoS_2_ and composites, (i) the Csp value depends on the morphology and SSA and (ii) the Csp retention is controlled by the VGCNF. On the other hand, supercapacitor exhibiting long term cyclic stability is also highly desirable. The evaluation of cyclic stability was performed at a high charge transfer of 200 mV s^−1^ as shown in Fig. 7f. The MPR25-1 electrode shows only 4% reduction after 1,000 cycles, demonstrating good long term stability which is much better than the bare MoS_2_, which shows 24% reduction. This is achieved due to the high charge storage and high electrochemical stability of MPR25-1 after the addition of VGCNF. On the other hand, MGO has a smaller Csp than MPR25-1 due to the lower SSA and conductivity. Compared to the published results listed in Table 2, we clearly demonstrate the potential application of these composites for supercapacitor application.

**Table 2:** Comparison of electrochemical performance of MoS_2_ composite electrodes.

No.	Electrode	Method	Csp	Retention	Refs
1.	MoS_2_-RGO	MAH for 60 min	265 F g^−1^ at 10 mV s^−1^	92% after 1000 cycles at 20 mV s^−1^	15
2.	MoS_2_-polyaniline-RGO	Hydrothermal at 180 °C for 48 h (MoS_2_ synthesis), hydrothermal at 180 °C for 12 h (RGO synthesis) + polymerization for 12 h	618 F g^−1^ at 1 A g^−1^	78% after 2000 cycles at 20 A g^−1^	26
3.	MoS_2_-polyaniline	Exfoliated MoS_2_ + polymerization for 6 h	390 F g^−1^	86% after 1000 cycles at 0.8 A g^−1^	17
4.	MoS_2_-NDG	Hydrothermal at 180 °C for 36 h	245 at 0.25 A g^−1^	91.3% after 1000 cycles at 2 A g^−1^	11
5.	MoS_2_-graphene	Hydrothermal at 180 °C 36 h	243 F g^−1^ at 1 A g^−1^	93.3% after 1000 cycles at 1 A g^−1^	16
6.	MoS_2_-polypyrrole	Hydrothermal at 200 °C for 24 h and polymerization for 12 h	553.7 F g^−1^ at 1 A g^−1^	90% after 500 cycles at 1 A g^−1^	10
7.	MoS_2_-VGCNF	MAH at 200 °C for 30 min	248 F g^−1^ at 5 mV s^−1^	96% after 1000 cycles at 200 mV s^−1^	This work

**Figure 8:**
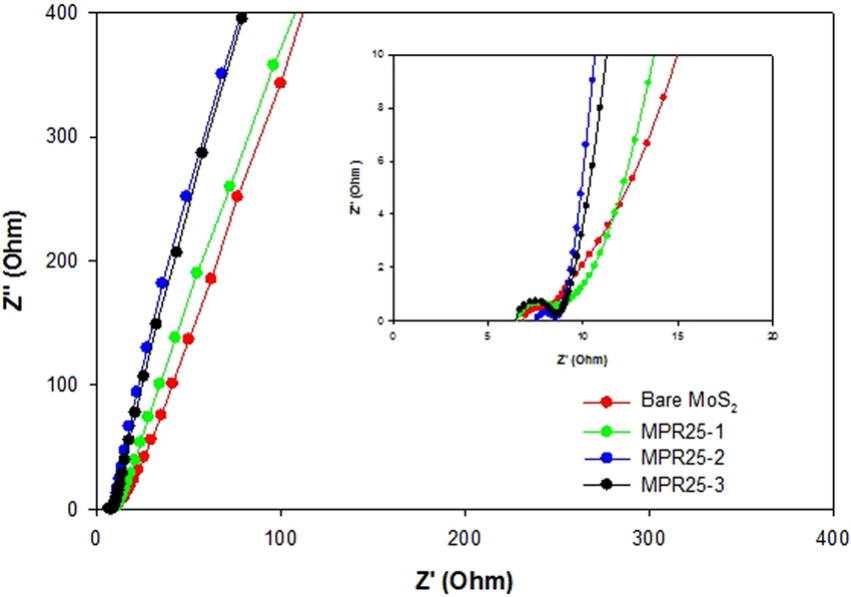
Nyquist plot of bare MoS_2_ and composites. The inset shows the magnification of the high-frequency region of the spectra.

To analyse the resistivity of supercapacitor cell, EIS test was performed and the resulting Nyquist plots are shown in Fig. 8. At the low frequency region, the slope indicates idealness of capacitor behaviour. It shows that the composites are closer to an ideal capacitor due to the presence of VGCNFs that improve the charge transfer. At the high frequency region, the first point at the X-axis is the equivalent series resistance (ESR/Rs) which is associated with the intrinsic resistance of electrode material and the bulk ionic resistance in electrolyte solution^42^. As the same electrolyte was used, the difference is from the intrinsic resistance of the electrode material. As shown in Table 3, the addition of the conductive VGCNF decreases the Rs, except MPR25-2. The electrical conductivity increases with the fiber amount. The unique structure of MoS_2_ nanowall-VGCNF composite with intimate interface contact between MoS_2_ layers and VGCNF provides a diffusion path for enhanced charge transfer, i.e., improved electrical conductivity of composites. For MPR25-2, the higher Rs is attributed to the fact that MPR25-2 has the highest strain among the composites, leading to the formation of such defects that reduce the electrical conductivity. The MGO also shows higher Rs than the bare MoS_2_ due to the abundant oxygen atoms and O-functional groups on the GO. Furthermore, VGCNF also decreases the contact resistance (Rc) which is the intercept of quasi-semicircle with the X-axis. Rc is associated with the contact among the constituents in the MoS_2_/VGCNF composite electrode, and between the electrode and current collector. For the contact between the electrode and the current collector, the presence of VGCNFs bridges the transfer of electrons at electrode/current collector interface. However, MPR25-3 has a larger Rc because its thickest MoS_2_ layers make the transfer of electrons through interface less straightforward. Similarly, MGO has the largest Rc due its abundant oxygen atoms and O-functional groups that hinder the transfer of electrons at the interface. For the ion diffusion within the pores (Rd), bare MoS_2_ has higher Rd than the composites. This is due to that the bare MoS_2_ has thick mesoporous wall (determined from its pore volume). The mesoporous walls affect the diffusion distance in a manner that a thinner and thicker mesoporous wall leads to smaller and longer diffusion distance, respectively^49^. For the composites, the Rd values are lower, implying their mesoporous walls are thinner than that of the bare MoS_2_. Therefore, the diffusion distance is lower, favouring the electron transport. Moreover, the unique composite structure exhibiting low degrees of aggregation is highly desirable for ions intercalation.

**Table 3:** EIS analysis of bare MoS_2_ and composites.

ID sample	Rs (ohm)	Rc (ohm)	Rd (ohm)
Bare MoS_2_	6.9	1.8	4
MPR25-1	6.5	1.7	2.2
MPR25-2	7.5	1.1	0.7
MPR25-3	6.4	2	1.1
MPR25-1-2	5.7	1.5	2.6
MGO	8.7	3.25	3.2

## Conclusion

A novel nanocomposite electrode material, namely, 2D MoS_2_ nanowall/VGCNF has been successfully synthesized through a facile MAH method. The presence of VGCNF controls the morphology of the MoS_2_ layer and inhibits or reduces the aggregation of MoS_2_. The growth of MoS_2_ on the carbon nanofiber surface was made to happen due to the use of an acid agent, HCl. Such a unique growth has been explain using a growth model. Excellent electrochemical performance of the composite was also obtained due to the presence of the VGCNFs. The VGCNFs improved both conductivity and charge transfer electron by showing smaller Rs and Rc.

## Methods

### Synthesis of MoS_2_/VGCNFs composites

Firstly, VGCNF was subjected to acid treatment in 1 M HNO_3_ at 90 °C for 6 h for surface cleaning and oxidation. MoS_2_/VGCNF composite was synthesized by first mixing 1.09 g of Mo precursor Na_2_MoO_4_.2H_2_O (J.T. Baker) and 1.35 g of S precursor CH_4_N_2_S (Thiourea) (Riedel-de Haen) in 35 mL of de-ionized (DI) water. The solution then was stirred for homogeneity. After that, the pH value of the solution was adjusted to 2 through the addition of a 37% HCl solution (Sigma Aldrich). Subsequently, different amounts of VGCNF (Pyrograf Products Inc., Ohio, USA) was added to the solution under sonication to disperse the VGCNF. The molar ratios of the Mo precursor to the VGCNF were 1:0, 6:1, 2:1, and 1:1. For the MAH synthesis, the mixture was transferred in to a 100 ml Teflon autoclave which was then placed in a microwave digestion (Speedwave Galaxy 4). Microwave irradiation was carried out at a power of 100 W. The temperature was set at 200 °C for 30 min with a ramp rate of 15 °C/min. The sample was collected by filtrating and washing the solution after the MAH process with DI water and ethanol. The precipitation then was dried in a vacuum oven at 50 °C for 12 h. Thus obtained samples are designed hereafter in as bare MoS_2_ (for Mo precursor/VGCNF = 1:0), MPR25-1 (6:1), MPR25-2 (2:1), and MPR25-3 (1:1). For comparison, GO was also used in this study using the same experimental condition, except without any VGCNF, and the resulting sample is designed as MGO. GO was prepared following a previous study using a modified Hummers’ method^42, 50^. Another set of experiments has been done following the condition for preparing MPR25-1 except that VGCNF were dispersed in DI-water following by the dissolution of Mo and S precursors under stirring without sonication. The obtained sample is designed as MPR25-1-2. Meanwhile, without being subject to the MAH, the aforementioned Mo precursor/VGCNF mixture was also dropped on a glass substrate and then dried in an oven at 60 °C for several minutes. The dried products were analyzed for the phase and morphology.

### Characterization

The crystalline phase was examined using X-ray diffraction (XRD, Rigaku X-Ray Diffractometer) with Cu-Kα radiation (λ = 0.15406 nm). The surface chemistry and bonding were investigated using X-Ray photoelectron spectroscopy (XPS, Electron Spectroscopy for Chemical Analysis, PHI 5000). The surface morphology and microstructure were investigated using scanning electron microscopy (SEM, JSM-6701F) with an acceleration voltage of 3 kV and transmission electron microscopy (TEM, JEOL-2100F CS STEM) with an acceleration voltage of 200 kV. The zeta potential was determined using zeta potential analyser (Malvern Nano zs MRK791-02). The specific surface area (SSA) and pore size distribution were determined using the Brunauer-Emmett-Teller (BET) and the Barrett–Joyner–Halenda (BJH) method, respectively.

### Fabrication of working electrode

To prepare the working electrode, 70 wt% of the active material and 30% of carbon black were first dispersed in 1 mL ethanol which was then deposited onto a 1 × 1 cm^2^ Advantech filter paper using a filtration method. The ethanol was removed by drying the resulting sample at 60 °C. The active material side of the sample was attached to a stainless steel current collector to form the working electrode. The cell also had a filtration membrane (Critical process) and a 1 M KCl aqueous solution electrolyte.

### Electrochemical measurements

All the electrochemical measurements were carried out at room temperature. The supercapacitor performance was evaluated in a three electrode cell for cyclic voltammetry (CV) measurement and a two electrode cell for electrochemical impedance spectroscopy (EIS) measurement using an Autolab PGSTAT. A three electrode cell consisting of a working electrode, a platinum counter electrode, and a saturated calomel electrode (SCE) reference electrode. CV measurement was performed within −0.9 to 0.4 V at scan rates from 5 to 100 mVs^−1^. The preparation of the electrode for the two-electrode cell or symmetric supercapacitor was same as that of the three-electrode cell. The specific capacitance (Csp) was calculated by first integrating the area under the CV curve to obtain the charge (Q in Coulombs), which was then divided by the mass of the active material (m in g), the scan rate (*v* in V s^−1^), and the potential window (V = Va − Vc in V) according to Eq. (5):

5 $$Csp=\frac{Q}{{\rm{\Delta }}V}=\frac{1}{2mv({V}_{a}-{V}_{c})}{\int }_{{V}_{a}}^{{V}_{c}}I(V)dV$$

where I(V) is the current as a function of voltage, and Va and Vc are anodic and cathodic potentials, respectively. EIS was used to evaluate the electrical resistivity of the cell. It was conducted in the frequency range between 100 kHz and 2 mHz with a perturbation amplitude of 5 mV versus the open-circuit potential.

## Electronic supplementary material

Supplementary Information
