# Supplementary material for: Direct Growth of MoS2 Nanowalls on Carbon Nanofibers for Use in Supercapacitor
Source: Sci Rep. 2017 Jul 20;7:5999. doi: 10.1038/s41598-017-05805-z (PMC5519755; doi:10.1038/s41598-017-05805-z)
Supplement: Supplementary file 1 — Supplementary Information [file 41598_2017_5805_MOESM1_ESM.pdf]

Supplementary Information

Direct Growth of MoS_2 Nanowalls on Carbon Nanofibers for Use in Supercapacitor

Fitri Nur Indah Sari, Jyh-Ming Ting*

Department of Materials Science and Engineering

National Cheng Kung University, Tainan, Taiwan

*Email: jting@mail.ncku.edu.tw

Table S1. D-spacing (002) plane ($d_{(002)}$) of MoS_2 layer and TEM

Sample	$d_{(002)}$ (nm)[a]	$d_{(002)}$ (nm)[b]
MoS_2	0.63	0.62
MPR25-1	0.65	0.64
MPR25-2	0.71	-
MPR25-3	0.76	0.74
MPR25-1-2	0.69	-
MGO	0.84	0.83

[a]Calculated from XRD

[b]Calculated from HR-TEM

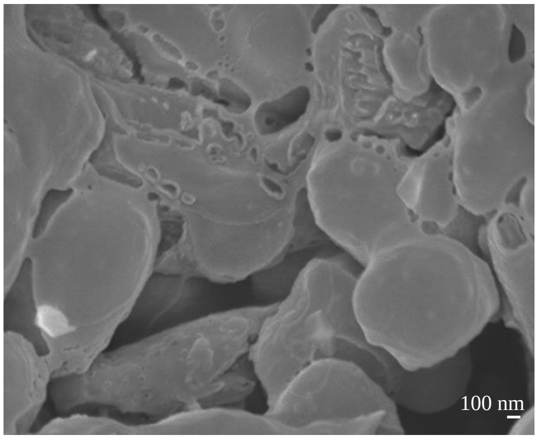

Figure S1. SEM image of Mo oxides nanoparticle

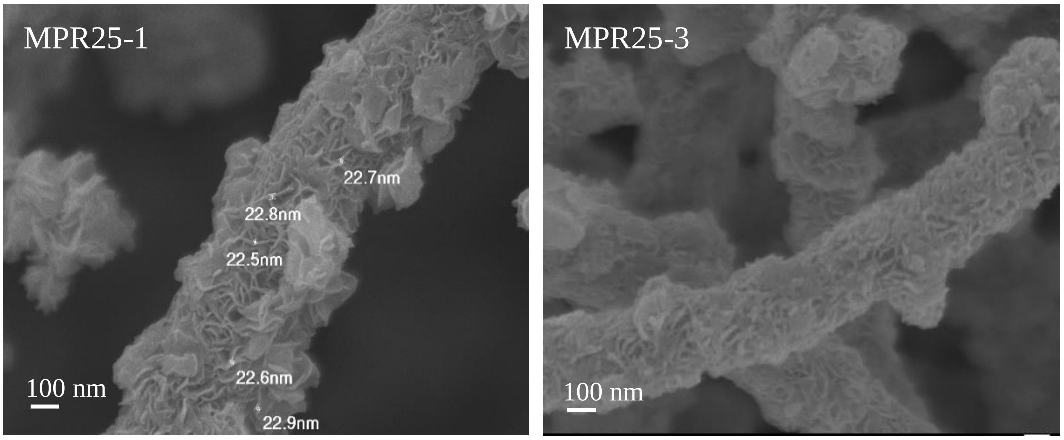

Figure S2. SEM images of MPR25-1 and -3.

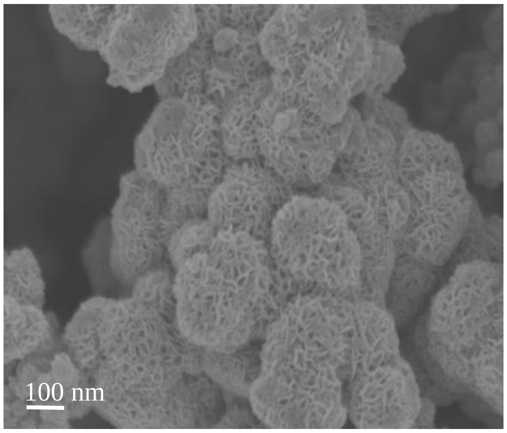

Figure S3. SEM image of MoS_2 nanoflowers by the RGO.

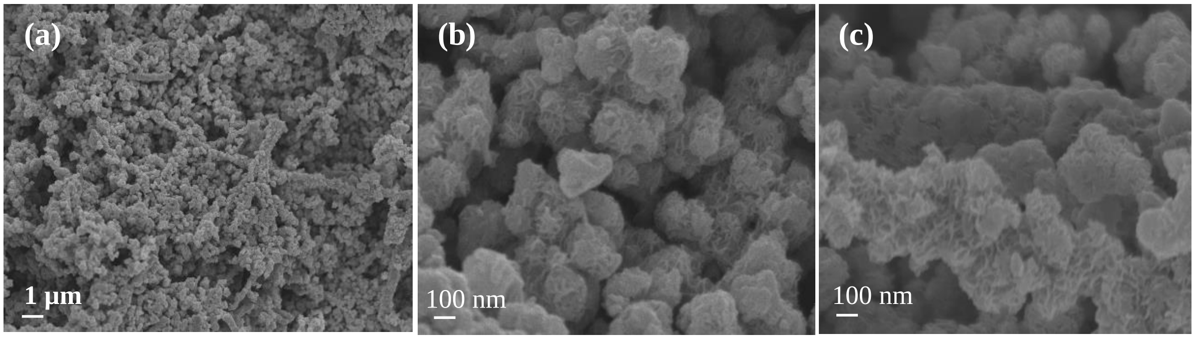

Figure S4. SEM images of MPR25-1-2. (a) Distribution of the composite, (b) MoS_2 nanoflowers by the fibers, and (c) MoS_2 nanowalls on the fibers.

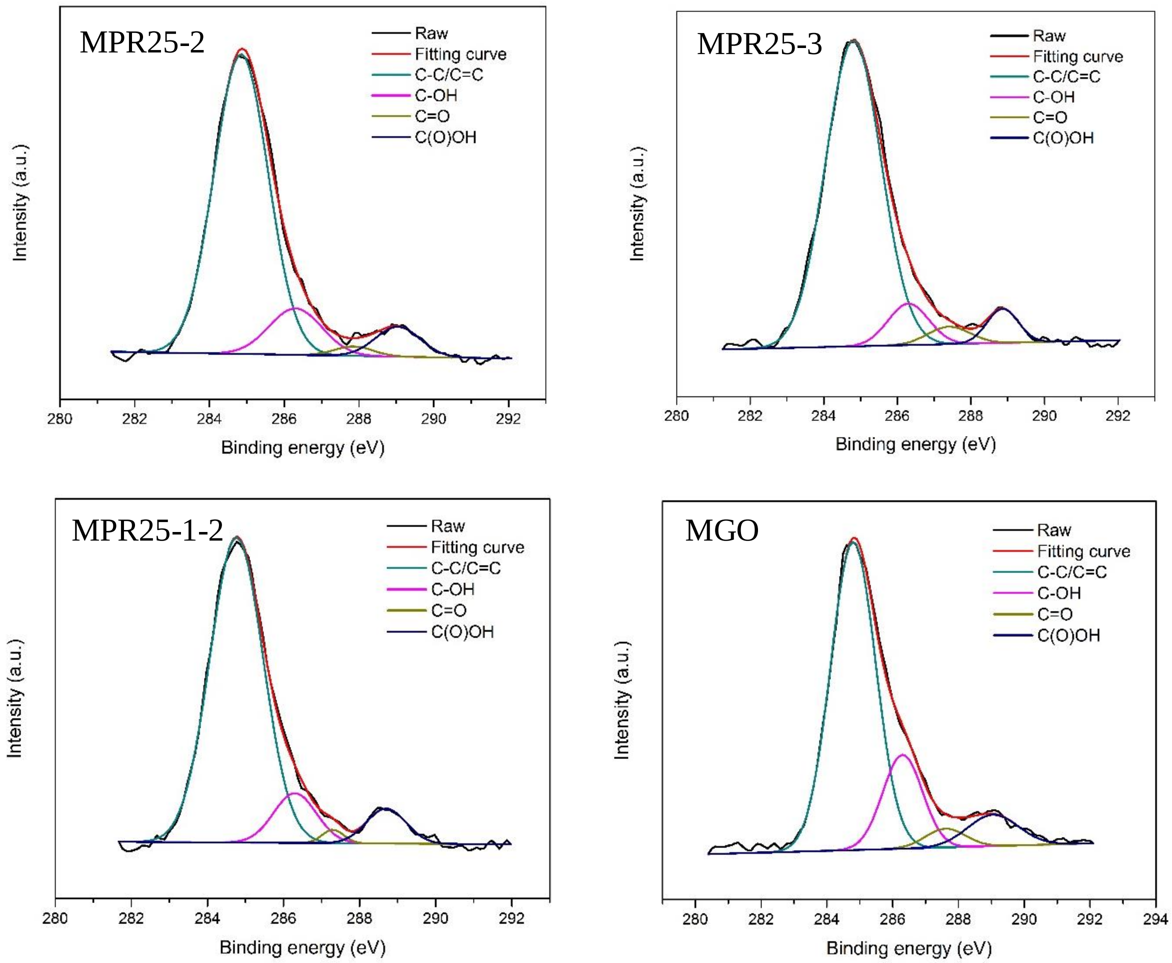

Figure S5. XPS C1s spectra of MoS_2 composites.

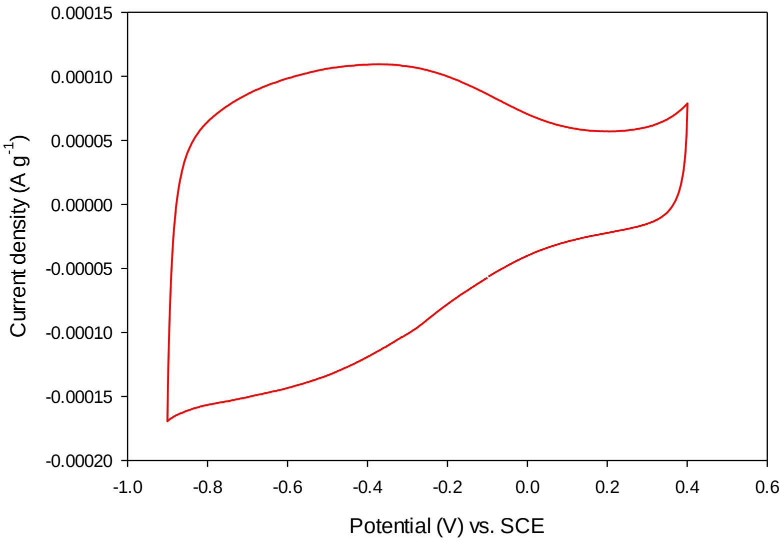

Figure S6. CV curve of VGCNF at 5 mV s^{-1}.

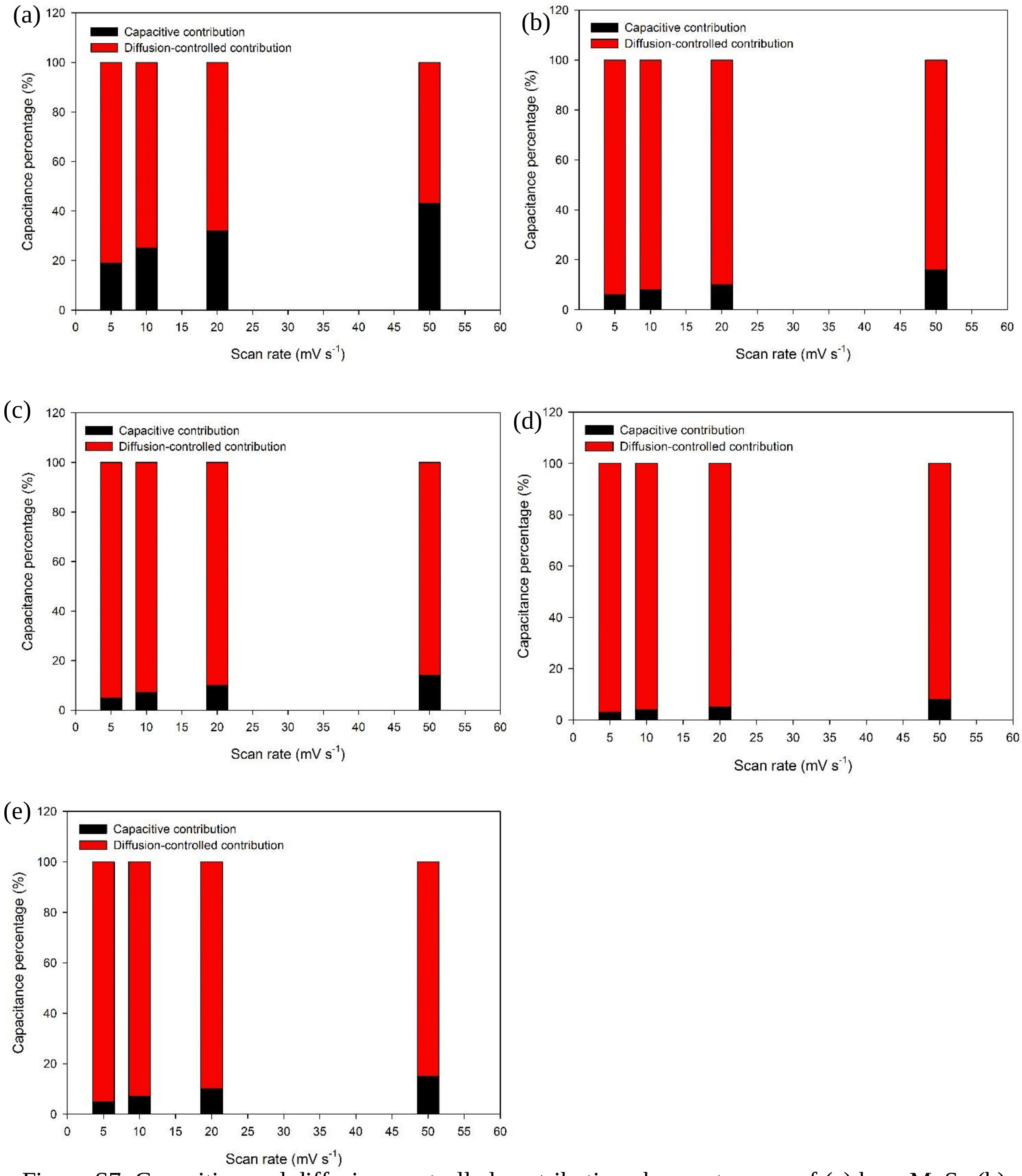

Figure S7. Capacitive and diffusion-controlled contribution charge storages of (a) bare MoS\, (b) MPR25-2, (c) MPR25-3, (d) MPR25-1-2, and (e) MGO at different scan rates.

Zeta Potential Measurement

The purpose was to show that the presence of NaCl would make the fiber surface charge becomes more positive. Therefore, the measurement was done through the addition of various amounts of NaCl into VGCNF-containing DI water. The result is shown in Figure S8. As shown in the figure, the surface charge of the VGCNF shifts positively with the NaCl.

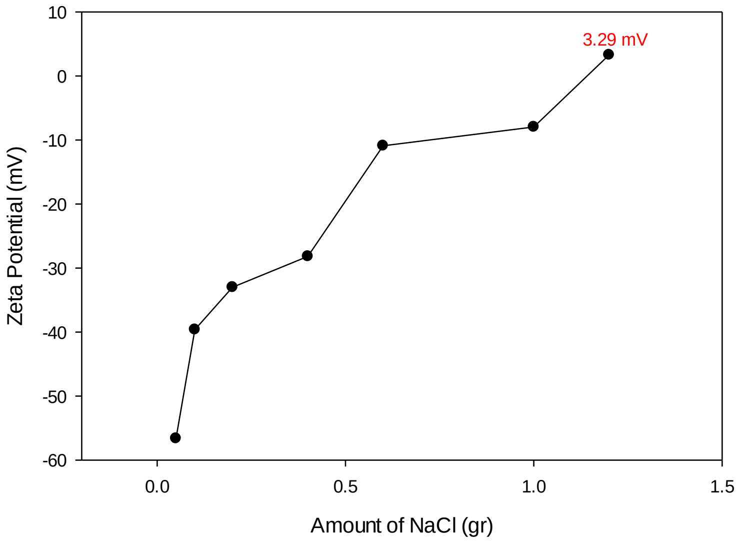

Figure S8. Zeta Potential value of VGCNF at different amount of NaCl.
